# Study of antibacterial mechanism of graphene oxide using Raman spectroscopy

**DOI:** 10.1038/srep28443

**Published:** 2016-06-21

**Authors:** Sitansu Sekhar Nanda, Dong Kee Yi, Kwangmeyung Kim

**Affiliations:** 1 Department of Chemistry, Myongji University, Yongin, 449-728, Korea; 2 KIST, Center for Theragnosis, Biomedical Research Institute, Seoul, 136791, Korea

## Abstract

Graphene oxide (GO) is extensively proposed as an effective antibacterial agent in commercial product packaging and for various biomedical applications. However, the antibacterial mode of action of GO is yet hypothetical and unclear. Here we developed a new and sensitive fingerprint approach to study the antibacterial activity of GO and underlying mechanism, using Raman spectroscopy. Spectroscopic signatures obtained from biomolecules such as Adenine and proteins from bacterial cultures with different concentrations of GO, allowed us to probe the antibacterial activity of GO with its mechanism at the molecular level. Escherichia coli (*E. coli*) and Enterococcus faecalis (*E. faecalis*) were used as model micro-organisms for all the experiments performed. The observation of higher intensity Raman peaks from Adenine and proteins in GO treated *E. coli* and *E. faecalis*; correlated with induced death, confirmed by Scanning electron Microscopy (SEM) and Biological Atomic Force Microscopy (Bio-AFM). Our findings open the way for future investigations of the antibacterial properties of different nanomaterial/GO composites using Raman spectroscopy.

The outer membrane of bacteria maintains their morphology and acts as a barrier of protection from external environments. It has, therefore, been a target for research in order to examine the antibacterial properties of different materials. Recently GO has attracted much attention as an antibacterial agent due its ability to induce oxidative stress by Reactive Oxygen Species (ROS) and lipid peroxidation. Hu *et al.*^1^ explained that the sharp edges of GO interfere with the physiological activity of bacteria, whereas Kotchey *et al.*^2^ proposed that the superoxide anion generated by GO can disrupt the membrane of bacteria. Later Akhavan *et al.*^3^,^4^,^5^ performed a series of experiments to demonstrate the behaviour of *E. coli* bacteria in the presence of GO. They showed that GO nanosheets with oxygen-containing functional groups can better trap the bacteria compared to graphene nanosheets with reduced functional groups^3^,^4^. They also demonstrated disruption of the cell membrane when it comes in direct contact with the sharp edges of GO nanowalls^5^. Liu *et al.* explained that the antibacterial effect of graphene derivatives was derived from oxidative stress that induces membrane disruption^6^. The most probable mechanism responsible for disruption of inner and outer cell membranes in *E. coli* has been proposed by Tu *et al.*^7^ According to this report, graphene nanosheets extract large amounts of phospholipids from the cell membranes due to strong dispersion interactions between graphene and lipid molecules. With the rapid development of Bionanotechnology, a great variety of GO composites are entering into our practical life. Most of them have been proven to possess antibacterial activity. So far, the exact mechanism at the molecular level has not been demonstrated. All of the above approaches can only monitor for morphological changes of *E. coli* induced by the presence of GO. To detect morphological changes in *E. coli* while co-cultured with GO, transmission electron microscopy (TEM) and scanning electron microscopy (SEM) are extensively used^7^. Leakage detection of intracellular contents, disorganization of cell membranes, and location of GO are common methods to explain the antibacterial properties of GO. Recently, Perreault *et al.*^8^ reported that the antimicrobial activity of GO increased up to 4-fold when GO surface area decreased from 0.65 to 0.01 μm^2^.

Scientist observed the strong antimicrobial properties of GO against a wide variety of both gram-positive and-negative bacterial pathogens, bio film forming microorganisms and phytopathogens^9^,^10^,^11^,^12^,^13^. Physical and chemical interactions between GO and bacterial cells were the major causes for antimicrobial properties of GO^5^,^14^. In this process, the bacterial cell membrane was observed to be a primary target for cytotoxicity study of GO. Membrane damage in GO exposed bacteria was identified by change in the transmembrane potential, morphological change in the cell structure, leakage of RNA and intracellular electrolytes, and uptake of membrane-impermeable dyes^7^,^10^,^15^. The sharp edges of GO allow it to penetrate through the cell membrane, and the consequential integrity disruption is the major cause of membrane damage^16^,^17^. The oxidative nature of GO also presents as another cause of membrane damage^11^,^12^.

Raman spectroscopy, as an important vibrational spectroscopic tool, can provide molecular fingerprint information on various chemical and biochemical components in complex systems like bacterial and cell cultures, biological tissues, etc.^18^,^19^,^20^,^21^. The application of Raman spectroscopy in a water-based medium without any complex sample pre-treatment is especially challenging. The changes in cellular composition such as in lipids, proteins, and nucleic acids can be monitored by Raman spectroscopy. However, due to the scattering behaviour of Raman photons the technique is less sensitive when the concentration of the materials is low. To overcome these problems, ultraviolet resonance Raman spectroscopy (UVRS) was used to achieve a selective enhancement of nucleic acid- and aromatic amino acid-related bands^22^,^23^.

In this paper we report on the antibacterial mechanism of GO at the molecular level via Raman spectroscopy at minimum inhibitory concentration (MIC). The detection of Raman bands associated with different biomolecules correlated to bacterial cell morphological changes has provided a clear image of the antibacterial effect of GO in cellular cultures of in *E coli* and *E. faecalis* at the molecular level. Specifically, with increasing GO concentration, Adenine and protein concentration also increases in the culture medium, an observation that is attributed to the rupture of the *E. coli* and *E. faecalis* cells. In other words, our work has further advanced the research of Tu *et al.*^7^ which indicated that excess release of Phospholipids from bacteria can induce bacterial death. Our findings assign the cause of bacterial death to the release of specific biomolecules, such as adenine and proteins. The major cause of this finding is the interaction between GO support and aromatic molecule through π-π stacking. An interaction occurs between polyaromatic domain and analytes on the surface of GO, which works as a suitable platform for molecular trapping. As a result, this helps to increase the Raman band intensity of GO. Induction of Raman band intensity will selectively help to unveil bacterial uptake mechanism and as to probe for bacterial diagnostics. Since, GO application towards antibacterial mechanism is comparatively new field, in near future our finding can be applied for biocatalyst, metabolite production. It will create new opportunity in the design of novel biosensors and measurement of several bioanalytical tools.

## Materials and Methods

### Materials

Graphite powder was purchased from Sigma-Aldrich Ltd. Potassium permanganate (KMnO_4_), sulphuric acid (H_2_SO_4_), hydrogen peroxide (H_2_O_2_), hydrochloric acid (HCl), were obtained from Dae Jung Chemicals and Metal Co., Ltd., South Korea. All the chemicals used in this research were research grade, and doubly distilled water was used throughout the experiments. All experiments are performed three times.

### Synthesis of GO

The synthesis of GO was carried out through a modification of Hummer’s method^24^. Briefly, a mixture of graphite flakes (2 g) and NaNO_3_ (1 g) was cooled to 0 °C in an ice bath. Concentrated H_2_SO_4_ (60 mL) was added to the mixture and then KMnO_4_ (9 g) was added slowly to keep the reaction temperature below 5 °C. When the addition of KMnO_4_ was completed, the reaction mixture was warmed to 35 °C and stirred for 7 h. The reaction mixture was then cooled to room temperature and poured into ice water (55 mL). Then, 7 mL of H_2_O_2_ was added, followed by the addition of 90 mL of ice water after separation. The reaction was subsequently stirred for 7 h and then subjected to filtration. After filtration, the product was washed three times with hydrochloric acid and then with deionized water for 120 h. All chemicals were purchased from Sigma-Aldrich except H_2_O_2_ that was purchased from Daejon chemicals.

### Characterization of GO

The morphology of GO was determined by SEM. The UV−vis spectra were obtained using a Varian UV−vis spectrometer. All of the spectra were normalized, according to the maximum absorption band value. The crystal structure and orientation was determined by a Rigaku X-ray diffractometer (XRD) operated at 40 KeV and 40 mA with Cu Kα radiation in the range of 10°−60° with a step of 0.02°. Bio-AFM studies were done by Nanowizard II, JPK, Germany.

### Test of Antibacterial Properties

The microdilution method was used with Gram- bacterial strains (*Escherichia coli* (KACC 10005) and Gram + bacterial strain (*E. faecalis*) to determine the antibacterial activity of GO^5^. Lysogeny broth (LB) was used as the diluent for both bacterial strains. Inoculates were prepared by suspending cells in sterile LB media for 12 h. GO and standards were placed in 96-well plates and 10^7^ CFU/mL of cells were inoculated so that the final volume in each microwell was 0.2 mL. The plates were incubated at 35 °C for 24 h and the absorbance was read at 590 nm using a microplate reader. MIC values were determined both before and after incubation. 1 mL of 24 h old bacterial suspension (approx. 10^8^ CFU) containing both *E. coli* and *E. faecalis* was inoculated in a petri dish and was incubated at 30°C with continuous shaking at 200 rpm for 18–24 h. Bacterial culture containing *E. coli* and *E. faecalis* was used as control. Kanamycin was used as a standard drug for both bacteria. After 24 h of incubation, images of the bacterial colonies were taken.

### Sample Preparation for Raman Spectra Measurements

*E. coli* cells were allowed to grow in LB medium until OD (optical density) reached to 0.17. Then the cells were centrifuged at 10000 rpm for 30 min to remove the LB media prior to addition of GO. Afterwards, cells were suspended in 1 mL of deionized water. Then different concentrations of GO were added to the washed bacteria cells to bring the final concentrations to 50 μg/mL, 100 μg/mL, 150 μg/mL in 1 mL of *E. coli* solution. After vortexing, an aliquot of 20 μL of the mixture was dropped onto a glass slide for the Raman spectra measurement. All experiments are performed three times.

### Raman Spectra Measurements

Raman spectra were acquired using a Reinshaw micro-Raman system equipped with a 1800/500 g/mm grating. Excitation was provided by a He−Ne 633 nm laser with a power of 70 μW shined on the sample. A 10× objective (Olympus) lens with a numerical aperture of 0.55 and a working distance of 8 mm was used to focus the laser beam and collect the Raman signal. An acquisition time of 2−10 s was used. Duo Scan in the micro mapping mode with a scanning area of 30 μm × 30 μm was used to reduce any possible laser damage to Bacteria.

### Effect of GO on the leakage of proteins from the membrane of *E. coli* and *E. faecalis*

150 μL of GO suspension was added to 100 mL of LB medium containing 10^8^ CFU/mL of *E. coli* and *E. faecalis* bacterial culture. The GO treated culture was then incubated at 30 ± 2 °C with continuous shaking at 200 rpm. Samples were collected from the GO treated bacteria every two hours. The collected samples were centrifuged at 12,000 rpm for 5 minutes, the supernatant was then collected and frozen at −20°C. Standard methods were used to determine the amount of proteins leaked^25^.

### Bacterial membrane peroxidase activity

TMB (3, 3′, 5, 5′-tetramethylbenzidine) assay was used for determination of enzymatic activity of bacteria^26^. 80 μL of TMB, 100 μL of GO and 20 μL of bacteria (10^9^/mL) were mixed thoroughly. Samples were incubated at 37 °C for 30 minutes with continuous shaking at 200 rpm. After the incubation period, 50 μL of 2 N H_2_SO_4_ was added to terminating TMB reaction and the OD of the resulting blue color was read at 450 nm using a PerkinElmer Victor 3 spectrophotometer (PerkinElmer Inc., Waltham, MA, USA).

## Results and Discussion

### Characterisation of GO

**Figure 1:**
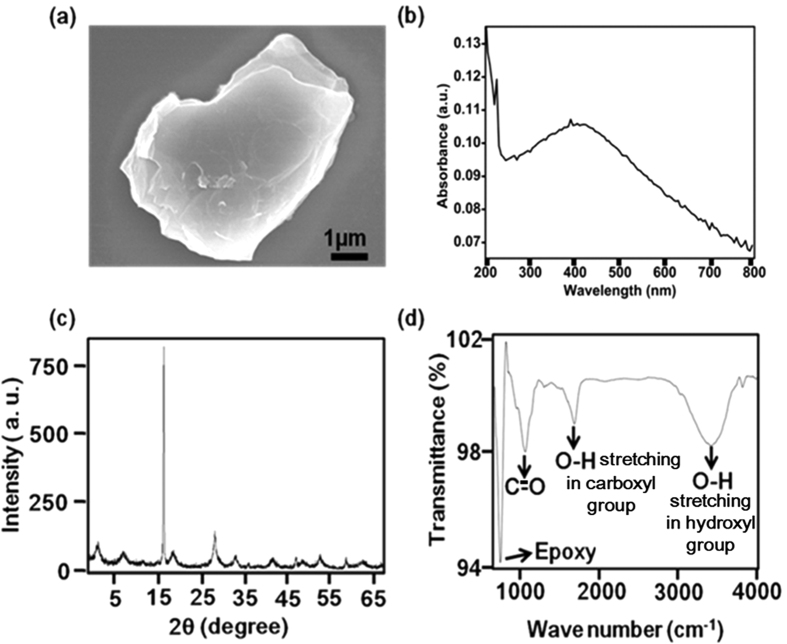
(**a**) SEM images of GO at a scale bar of 1 μm (**b**) UV-Vis spectra of GO; the main absorbance peak shows at 232 nm. (**c**) XRD pattern of GO; the strong and sharp peak at 2θ = 15.10° corresponds to an interlayer distance of 7.15 Å (**d**) The FT-IR spectra of GO show the presence of hydroxyl, epoxy, carboxyl and carbonyl (C=O) groups.

In Fig. 1(a) typical field emission scanning electron microscopy (FE-SEM) images of GO are shown. In Fig. 1(b) the main absorbance peak attributable to π-π* transitions of C=C in synthesized GO occurrs at around 232 nm, which is similar with the explanation of Singh *et al.*^27^ In Fig. 1(c) the XRD spectra of GO show a distinct peak at 15.10° corresponding to a d-spacing (in this case, the interlayer distance between sheets) of approximately 7.15 Å that is due to interlamellar water trapped between hydrophilic GO sheets^28^,^29^. In Fig. 1(d) the Fourier transform infrared (FTIR) spectra of the GO clearly show the presence of carboxyl (O-H deformation 1730-1700 cm^−1^), hydroxyl (O-H stretching vibration 3450 cm^−1^), epoxy (750 cm^−1^), and carbonyl (C-O stretching 1050 cm^−1^) groups. Si *et al.* obtained similar data for FTIR spectra^30^.

**Figure 2:**
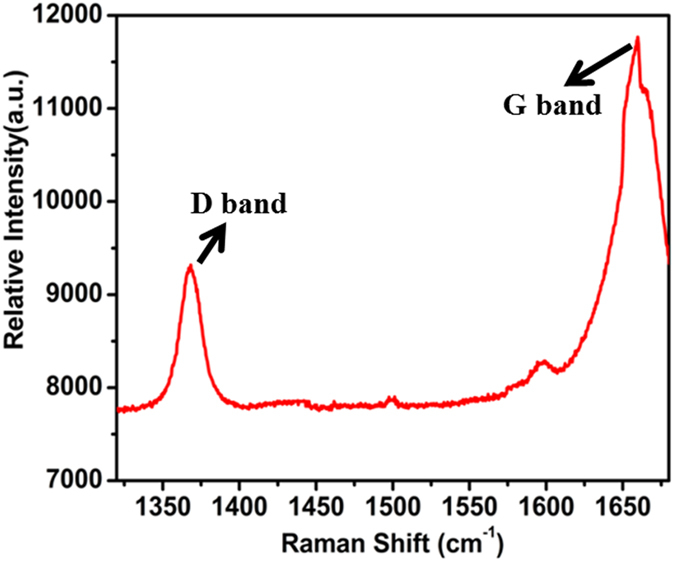
Raman Spectrum of GO with a 633-nm laser excitation wavelength. The G-band and D-band of GO appear at 1660 cm^−1^ and 1380 cm^−1^, respectively. The G-band arises from the stretching of the C-C bond in graphitic materials, and is common to all sp^2^ carbon systems. The D-band arises from the structural imperfections created by the attachment of hydroxyl and epoxy groups on the GO basal plane.

Raman spectroscopy affords a non-destructive technique in the study of the bonding nature of graphite material such as different carbon nanotubes (CNT), GO and graphene^31^. Herewith, firstly Raman spectroscopy was employed to study the disorder and defect levels of GO, and secondly the antibacterial properties of GO were examined. In general, the Raman spectrum of graphite exhibits a ‘G band’ at 1580 cm^−1^ and a ‘D band’ at 1350 cm^−1^. The G band is due to the first order scattering of the E_2_ _g_ mode whereas the D band is related to the defect in the graphite lattice^32^. The Raman spectra of GO are shown in Fig. 2, which show the presence of a G band at 1660 cm^−1^ and a D band at 1380 cm^−1^. The G band of GO is shifted towards a higher wave number, an observation that co-relates with the oxidation of graphite which results in the formation of sp^3^ carbon atoms. Furthermore, the D band in the GO is broadened due to the size reduction of the in-plane sp^2^ domains during oxidation^33^.

### Study of the antibacterial activity of GO and its underlying mechanism

**Figure 3:**
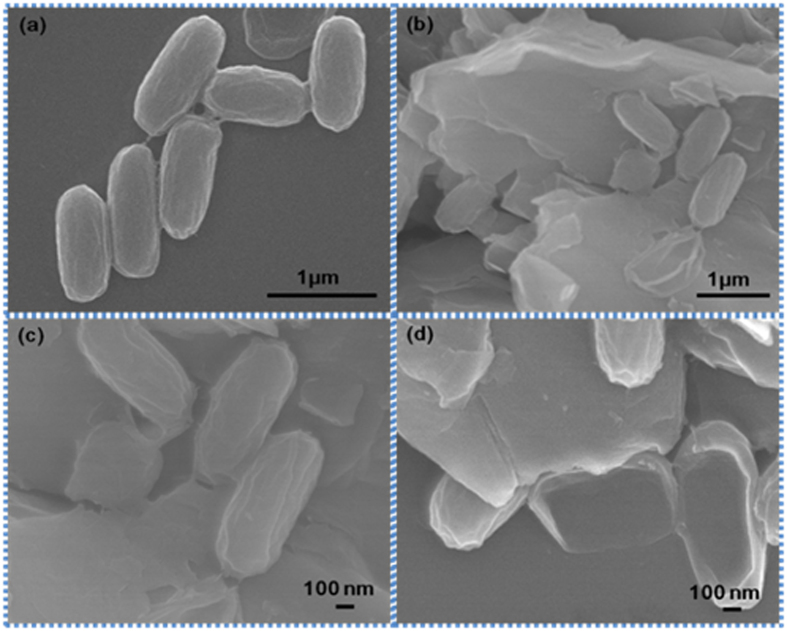
Antibacterial activity of GO followed by TEM imaging. (**a**) Images of *E. coli* (control) (**b**) *E. coli* treated with GO (**c,d**) Enlarged views of *E. coli* treated with GO. The size and shape of the *E.coli* bacteria differ when treated with GO.

**Figure 4:**
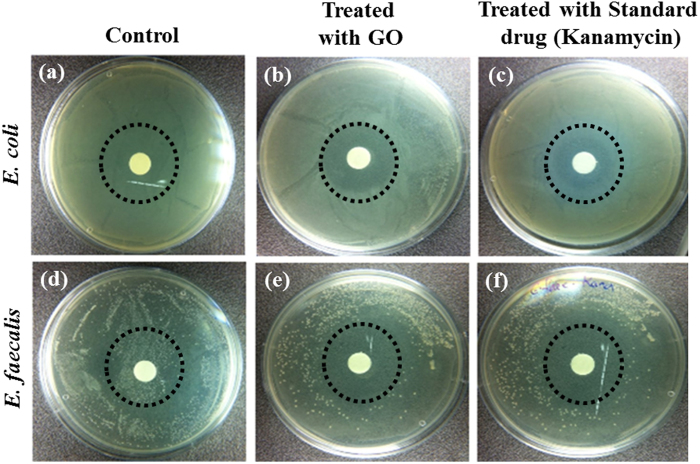
Photographs of bacterial colonies formed by *E. coli* and *E. faecalis*. (**a**) *E. coli* (control) (**b**) *E. coli* treated with GO (**c**) *E. coli* treated with standard drug (Kanamycin) (**d**) *E. faecalis* (control) (**e**) *E. faecalis* treated with GO (**f**) *E. faecalis* treated with standard drug (Kanamycin).

**Figure 5:**
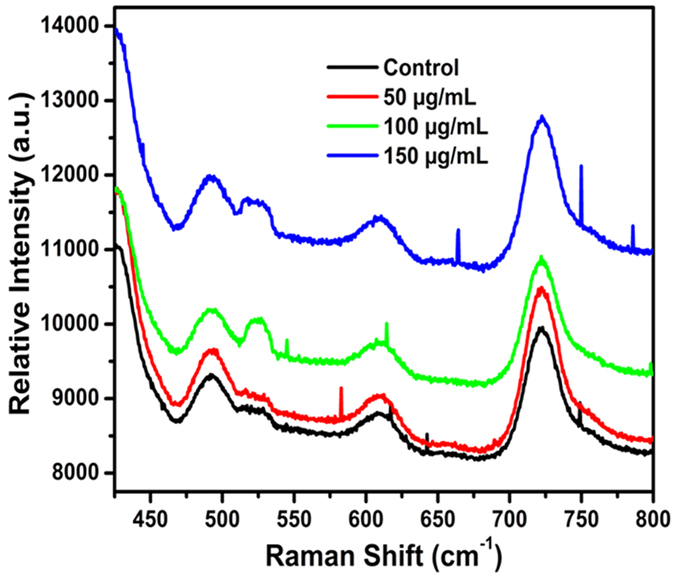
Raman spectra of GO treated *E. coli*; (control-black line) and different GO concentrations (50 μg/mL-red line, 100 μg/mL-green line, and 150 μg/mL-blue line). The data show an increment of Adenine (729 cm^−1^), and proteins (S-S stretching vibration at 490 cm^−1^ and Amide VI band at 610 cm^−1^) concentration in *E. coli* when GO concentration increases.

The antibacterial activity of GO was determined by the microdilution method [5] and the MIC value measured against *E. coli* was 1 μg/mL. Simultaneously, the MIC value measured against *E. faecalis* was 4 μg/mL. In Fig. 3(a) *E. coli* images are shown. In Fig. 3(b) *E. coli* treated with GO images are shown, while enlarged images are shown in Fig. 3(c). Similar images of *E. coli* with different shape are shown in Fig. 3(d). The antibacterial effects were investigated. Representative photos in Fig. 4 shows the GO induced inhibition zones for both *E. coli* and *E. faecalis*. We speculate that GO possess antibacterial property that inhibit the growth of both Gram− and Gram+ bacterial cells. In order to better understand the antibacterial mechanism of GO based on changes in Raman bands, it is important to know their molecular origins. Among the bands displaying obvious changes, the 729 cm^−1^ band was assigned to Adenine^34^. As the concentration of GO increased, the concentration of the Adenine ring mode increased, as shown in Fig.5.

In order to maintain a given protein conformation, disulphide bonds are always present on the surface of Bacteria. The conformation about these bonds is related to the structure of the protein. The S-S stretching vibration occurred at 490 cm^−1^ as shown in Fig. 5. The intensity of the S-S stretching vibrations increased as the concentration of GO increased. Different conformational arrangements about the disulphide bond lead to the S-S stretching vibration bands occurring in different positions. The spectra of all proteins exhibit absorption bands due to their characteristic amide group, CO-NH. Hence, the characteristic bands of the amide group of protein chains are similar to those of ordinary secondary amides. The bands of proteins are labelled in the same way as amide bands. This is in order to reflect the various contributions to the bands made by the vibrations^34^,^35^. Here, the Amide VI band (CO-NH bending vibration) occurred at 610 cm^−1^ which is shown in Fig. 5. The intensity of Amide VI band (CO-NH bending vibration) increased as the concentration of GO increased. Finally, by increasing the concentration of GO in the *E. coli* culture the Adenine and proteins band intensity increased leading to *E. coli* bacterial death. Similar results were found in the case of *E. faecalis.*

### Morphology

**Figure 6:**
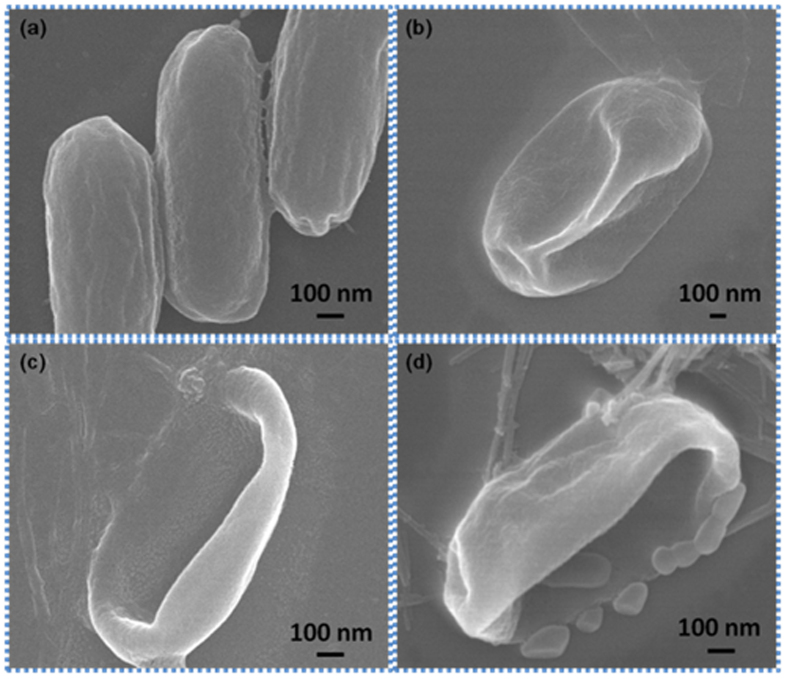
(**a**) SEM images of *E. coli* (control) (**b**) SEM images of *E. coli* when treated with 50 μg/mL of GO (**c**) SEM images of *E. coli* when treated with 100 μg/mL of GO (**d**) SEM images of *E. coli* when treated with 150 μg/mL of GO. As the concentration of GO increases the shape of *E. coli* changes.

In Fig. 6 SEM images of *E. coli* bacteria and the effect of its treatment with GO are observed. As the concentration of GO increased morphological changes in the shape of the *E. coli* bacteria were observed. Here, GO penetrate cellular membrane of *E. coli* through its sharp edges. The clear membrane damages are observed in Fig. 6. In Fig. 6(a) SEM images of *E. coli* (control) observed. In Fig. 6(b) SEM images of *E. coli* showed that small changes in shape of bacteria. In this experiment 50 μg/mL of GOs was treated with *E. coli*. In Fig. 6(c) SEM images of *E. coli* showed that penetration of GO into bacteria. In this experiment 100 μg/mL of GOs was treated with *E. coli*. In Fig. 6(d) SEM images of *E. coli* showed that intracellular leakage from bacteria. In this experiment 150 μg/mL of GOs was treated with *E. coli*. As previously described, the shape of the *E. coli* bacterium influences where it attaches to GO; the same effect was found here as well.

**Figure 7:**
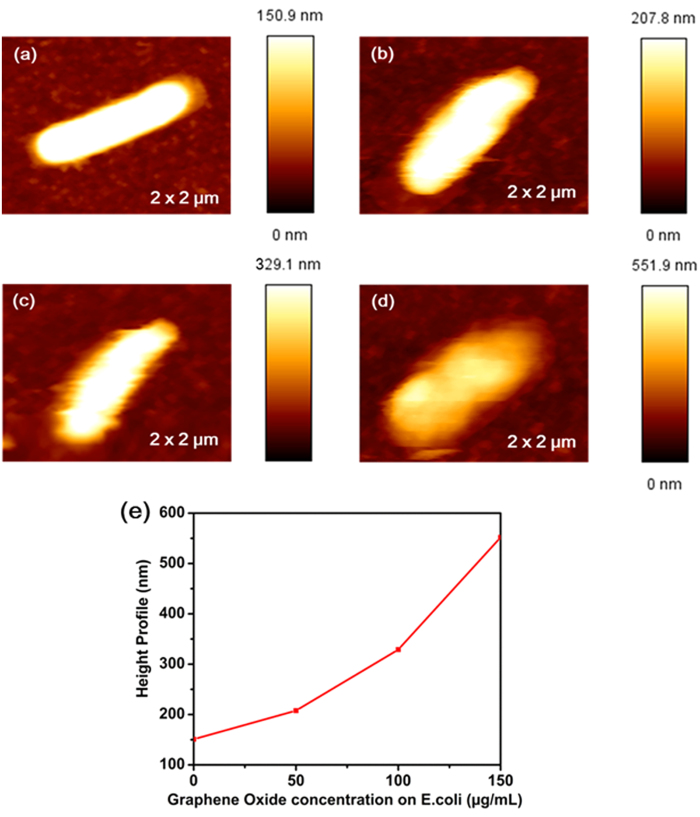
Bio- AFM images of *E. coli* cells, before and after treatment with various concentrations of GO, immobilized on a glass slide. (**a**) Images of untreated *E. coli*, showing a height profile of 150.9 nm. (**b**) Images of *E. coli* after GO of 50 μg/mL concentration was added showing an increased height profile up to 207.8 nm. (**c**) Images of *E. coli* after GO of 100 μg/mL concentration was added showing an increased height profile up to 329.1 nm. (**d**) Images of *E. coli* after GO of 150 μg/mL concentration was added showing an increased height profile up to 551.9 nm. (**e**) Plot of bacterial height profile (y-axis) vs. GO concentration (x-axis); as the concentration of GO increased the *E. coli* height profile increased.

**Figure 8:**
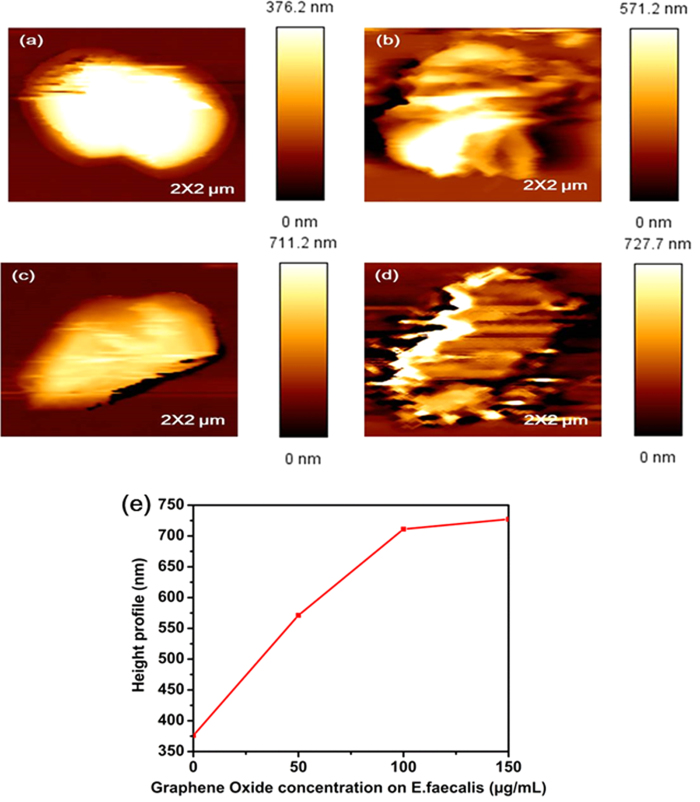
Bio- AFM images of *E. faecalis* cells, before and after treatment with various concentrations of GO, immobilized on a glass slide. (**a**) Images of untreated *E. faecalis*, showing a height profile of 376.2 nm. (**b**) Images of *E. faecalis* after GO of 50 μg/mL concentration was added showing an increased height profile up to 571.2 nm. (**c**) Images of *E. faecalis* after GO of 100 μg/mL concentration was added showing an increased height profile up to 711.2 nm. (**d**) A concentration of 150 μg/mL of GO was added to images *E. faecalis* after GO of 150 μg/mL concentration was added showing an increased height profile up to 727.7 nm. (**e**) Plot of bacterial height profile (y-axis) vs. GO concentration (x-axis); as the concentration of GO increased the *E. faecalis,* height profile increased.

The morphology of bacteria was studied to better understand the influence of GO on bacterial cell structure. Gram– bacteria (*E. coli)* composed of a thin peptidoglycan layer (7–8 nm thickness) and Gram + bacteria (*E. faecalis*) composed of a thick peptidoglycan layer (20–80 nm thickness)^5^ were studied. In Fig. 7(a) the height profile of *E. coli* is observed to be 150.9 nm and in Fig. 8(a) the height profile of *E. faecalis* is 376.2 nm. Figure 7 confirmed that as the concentration of GO increased on *E. coli*, the height profile increased. When concentrations of 50 μg/mL, 100 μg/mL, 150 μg/mL of GO were added to *E. coli,* the height profile became 207.8 nm, 329.1 nm, 551.9 nm, respectively. Figure 8 confirmed that as the concentration of GO increased in *E. faecalis* bacterial cultures, the height profile also increased. When 50 μg/mL, 100 μg/mL, 150 μg/mL of GO were added to *E. faecalis*, the height profile became 571.2 nm, 711.2 nm and 727.7 nm, respectively. From this observation we conclude that the antibacterial mechanisms for Gram + and Gram– bacteria are identical. In addition, it was clearly observed that when the concentration of GO is increased, degradation of the outer and the inner cell membranes of both Gram + bacteria and Gram– bacteria is induced.

**Figure 9:**
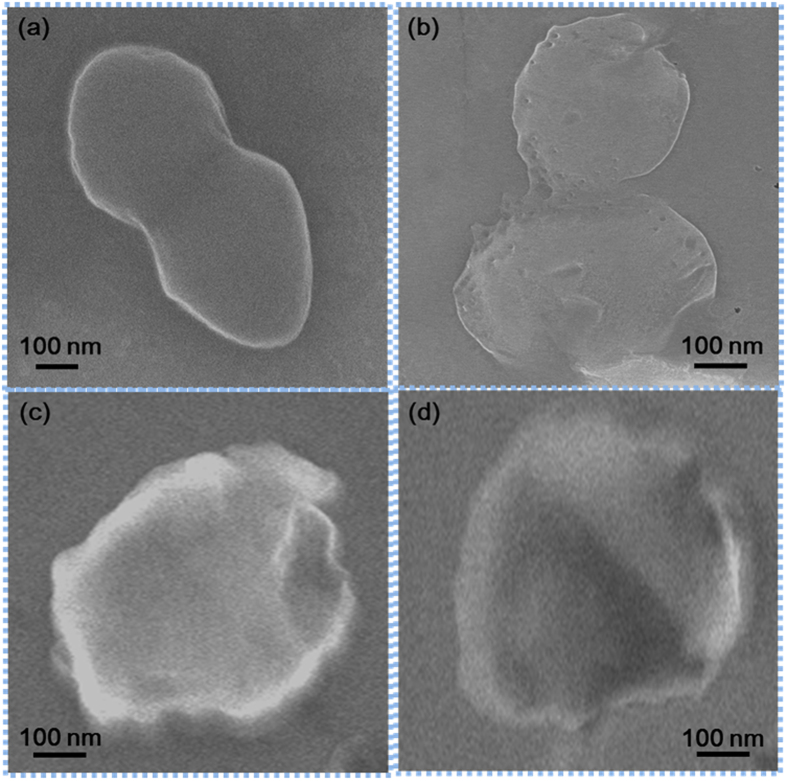
SEM images of *E. faecalis* before and after treatment with GO (a) control (b) 50 μg/mL GO added (c) 100 μg/mL GO added and (d) 150 μg/mL GO added. As the concentration of GO increases *E. faecalis* is seen to acquire different shapes. The images were observed under SEM.

**Figure 10:**
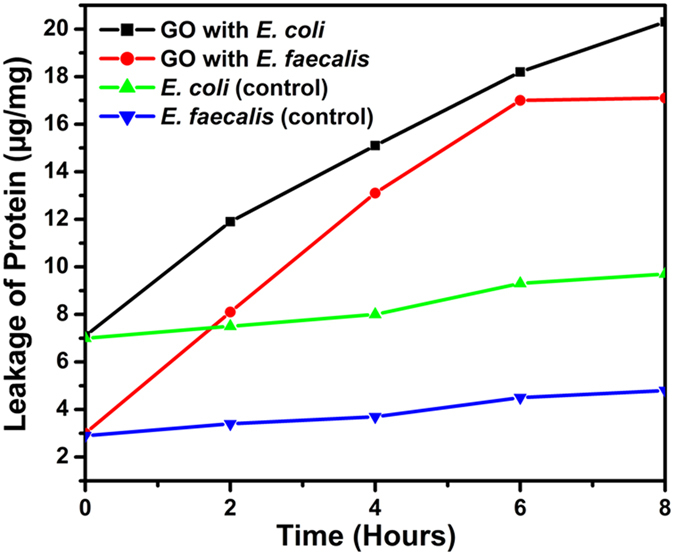
Leakage of proteins from *E. coli* and *E. faecalis* microorganism. As the incubation time increased, the leakage of protein increased.

In Fig. 9 SEM image of *E. faecalis* before and after treatment with GO are shown. The appearance of structural or morphological changes in *E. faecalis* with increasing GO concentration was confirmed. The leakage of proteins from both *E. coli* and *E. faecalis* were quantified as illustrated in Fig. 10. It was observed that as the incubation time increased, the leakage of protein from both *E. coli* and *E. faecalis* increased. Initially 7.1 and 3.0 μg/mg proteins were leaked from *E. coli* and *E. faecalis,* respectively. The protein content was measured every 2 h for 8 h, the amount of leaked protein was found to increase to 11.9, 15.1, 18.2, 20.3 μg/mg for *E. coli* and 8.1, 13.1, 17.0, 17.1 μg/mg for *E. faecalis*, respectively. From these results, it can be concluded that GO is more effective against Gm- bacteria (*E. coli*) as compared to Gm + bacteria (*E. faecalis*). This result is well matched with our previous finding.

**Figure 11:**
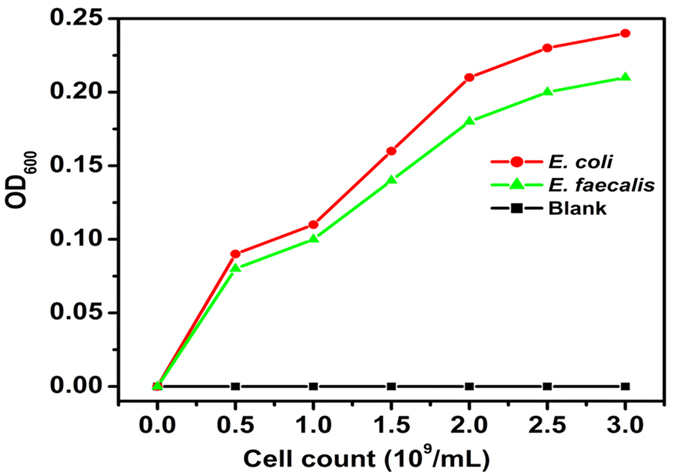
Bacterial membrane peroxidase activity by using TMB as a substrate. *E. coli* showed higher enzymatic activity due to its thin peptidoglycan layer as compared with *E. faecalis*.

**Figure 12:**
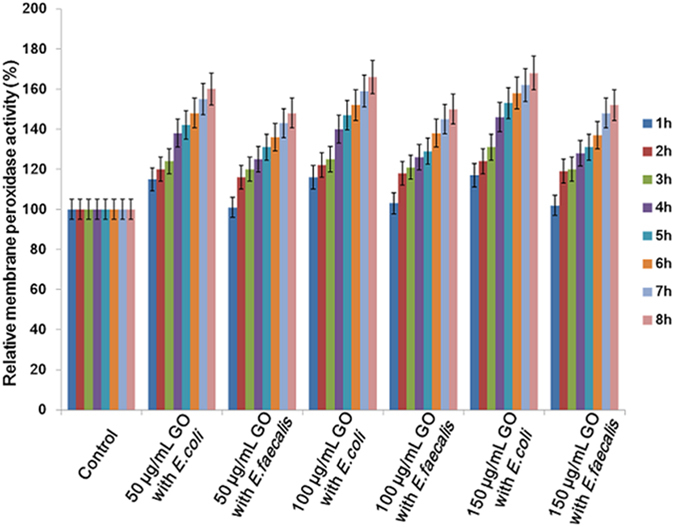
Specific membrane peroxidase activity of *E. coli* and *E. faecalis* in the presence of GO (50 μg/mL, 100 μg/mL, 150 μg/mL) compared to control (0 μg/mL) cells. Higher membrane peroxidase activities were found in time and dose depending manner for *E. coli* cells.

TMB was used as a substrate for membrane peroxidase activity study. In Fig. 11 showed that the enzymatic activity between GO and bacteria was proportional to the increase of the bacterial number. As we discussed previously, *E. coli* possess thinner peptidoglycan layer compare to *E. faecalis*. The higher enzymatic activity induced by GO to *E. coli* concluded that the sharp edges of GO allowing it to easily penetrate through *E. coli*’s thin peptidoglycan layer. Figure 12 depicts that both *E. coli* and *E. faecalis* possess higher membrane peroxidase activity in dose and time dependent manner compared with controlled cells. Moreover *E. coli* showed higher peroxidase activity than that of *E. faecalis*. This result supports our previous finding also. Here, TMB attracted towards negative charged GO via electrostatic interaction and a suitable change occurs when enzyme is coordinated with GO.

## Conclusions

Herewith, by using morphological and spectroscopic data we confirmed experimentally and theoretically that GO can induce the degradation of the outer and inner cell membranes of *E. coli* and *E. faecalis* bacteria. The work presented here demonstrates the great antibacterial action of GO is due to the release of Adenine and protein from Bacteria. Herein, we reported the exact mechanism at molecular level. The strategy can either be the use of GO as a Raman signal enhancer or the designed incorporation of GO in the novel antibiotics and other clinical applications. Antibacterial effects, bacterial inactivation and bacteriostatic effect of GO can be analysed by adjusting Raman signal. In future work, the size effect of GO on cytotoxicity will be investigated using Raman signal enhancer under relevant conditions. This will promote the use of GO in medical applications such as therapy, drug delivery and bio imaging in greater ways. Finally, the identified destructive extraction of Adenine and proteins offer a novel mechanism for GO antibacterial activity. Our finding will play a vital role in the design of new GO based antimicrobial materials.

## Additional Information

**How to cite this article**: Nanda, S. S. *et al.* Study of antibacterial mechanism of graphene oxide using Raman spectroscopy. *Sci. Rep.* **6**, 28443; doi: 10.1038/srep28443 (2016).
